# 1-(4-Chloro­butano­yl)-3-(3-chloro­phen­yl)thio­urea

**DOI:** 10.1107/S1600536814009295

**Published:** 2014-05-17

**Authors:** Hamza M. Abosadiya, Siti Aishah Hasbullah, Bohari M. Yamin, Adibatul H. Fadzil

**Affiliations:** a School of Chemical Sciences and Food Technology, Universiti Kebangsaan Malaysia, 43600 Bangi, Selangor D.E., Malaysia; b Low Carbon Research Group, School of Chemical Sciences and Food Technology, Universiti Kebangsaan Malaysia, 43600 Bangi, Selangor D.E., Malaysia; c Faculty of Applied Sciences, Universiti Teknologi MARA (UiTM), 40450 Shah Alam, Selangor D.E., Malaysia

## Abstract

The two independent mol­ecules in the asymmetric unit of the title compound, C_11_H_12_Cl_2_N_2_OS, exhibit different conformations, with the benzene ring and the N_2_CS thio­urea group forming dihedral angles of 87.40 (18) and 69.42 (15)°. An intra­molecular N—H⋯O hydrogen bond is present in each mol­ecule. Two further N—H⋯O hydrogen bonds link the independent mol­ecules into a dimer. In the crystal, the dimers are linked by N—H⋯S and C—H⋯S hydrogen bonds, forming chains parallel to the *c* axis.

## Related literature   

For applications and biological activities of thio­urea derivatives, see: Abbas *et al.* (2013 ▶). For the crystal structure of a related compound, see: Yusof *et al.* (2012 ▶). For bond-length data, see: Allen *et al.* (1987 ▶).
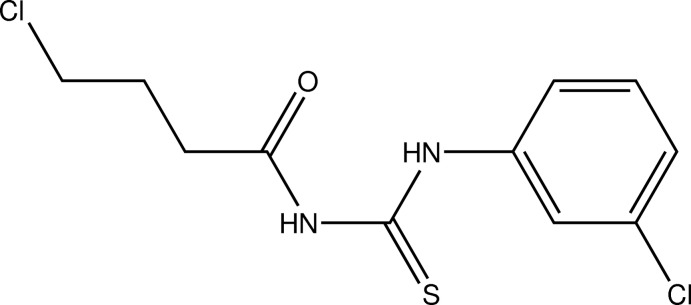


## Experimental   

### 

#### Crystal data  

C_11_H_12_Cl_2_N_2_OS
*M* *_r_* = 291.19
Monoclinic, 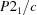

*a* = 14.7762 (8) Å
*b* = 10.9400 (6) Å
*c* = 17.8153 (10) Å
β = 111.327 (2)°
*V* = 2682.7 (3) Å^3^
*Z* = 8
Mo *K*α radiation
μ = 0.62 mm^−1^
*T* = 296 K
0.41 × 0.35 × 0.30 mm

#### Data collection  

Bruker SMART APEX CCD area-detector diffractometer
Absorption correction: multi-scan (*SADABS*; Bruker, 2009 ▶) *T*_min_ = 0.784, *T*_max_ = 0.835
49874 measured reflections
4987 independent reflections
3897 reflections with *I* > 2σ(*I*)
*R*_int_ = 0.046

#### Refinement  

*R*[*F* ^2^ > 2σ(*F* ^2^)] = 0.058
*wR*(*F* ^2^) = 0.162
*S* = 1.05
4987 reflections
323 parameters
4 restraints
H atoms treated by a mixture of independent and constrained refinement
Δρ_max_ = 1.51 e Å^−3^
Δρ_min_ = −0.83 e Å^−3^

### 

Data collection: *SMART* (Bruker, 2009 ▶); cell refinement: *SAINT* (Bruker, 2009 ▶); data reduction: *SAINT*; program(s) used to solve structure: *SHELXS97* (Sheldrick, 2008 ▶); program(s) used to refine structure: *SHELXL97* (Sheldrick, 2008 ▶); molecular graphics: *SHELXTL* (Sheldrick, 2008 ▶); software used to prepare material for publication: *SHELXTL* and *PLATON* (Spek, 2009 ▶).

## Supplementary Material

Crystal structure: contains datablock(s) global, I. DOI: 10.1107/S1600536814009295/rz5121sup1.cif

Structure factors: contains datablock(s) I. DOI: 10.1107/S1600536814009295/rz5121Isup2.hkl

Supporting information file. DOI: 10.1107/S1600536814009295/rz5121Isup3.cml

CCDC reference: 999317

Additional supporting information:  crystallographic information; 3D view; checkCIF report

## Figures and Tables

**Table 1:** Hydrogen-bond geometry (Å, °)

*D*—H⋯*A*	*D*—H	H⋯*A*	*D*⋯*A*	*D*—H⋯*A*
N2—H2⋯O1	0.89 (4)	1.97 (4)	2.679 (4)	136 (3)
N2—H2⋯O2	0.89 (4)	2.37 (3)	3.089 (4)	139 (3)
N4—H4⋯O1	0.88 (3)	2.38 (3)	3.106 (4)	140 (3)
N4—H4⋯O2	0.88 (3)	1.97 (3)	2.656 (4)	134 (3)
N1—H1⋯S2^i^	0.88 (3)	2.57 (2)	3.425 (3)	167 (3)
N3—H3⋯S1^ii^	0.87 (2)	2.54 (2)	3.397 (3)	169 (3)
C3—H3*B*⋯S1^iii^	0.97	2.87	3.792 (3)	160

Symmetry codes: (i) 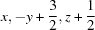; (ii) 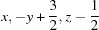; (iii) 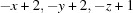.

**Fig. 1.**
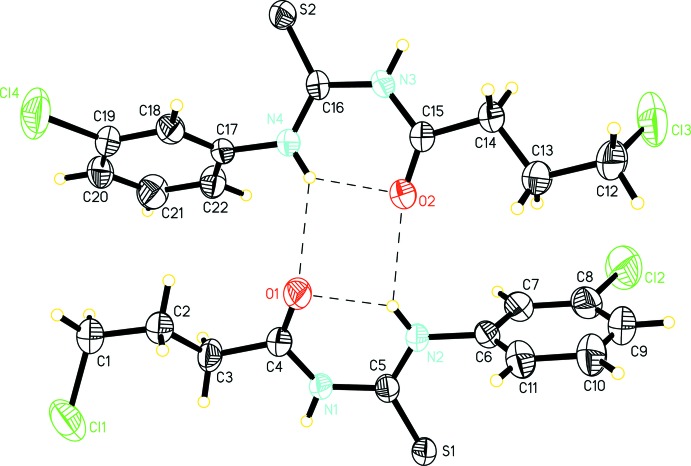
The asymmetric unit of the title compound with displacement ellipsoids drawn at the 50% probability level. The dashed lines indicate intermolecular hydrogen bonds.

**Fig. 2.**
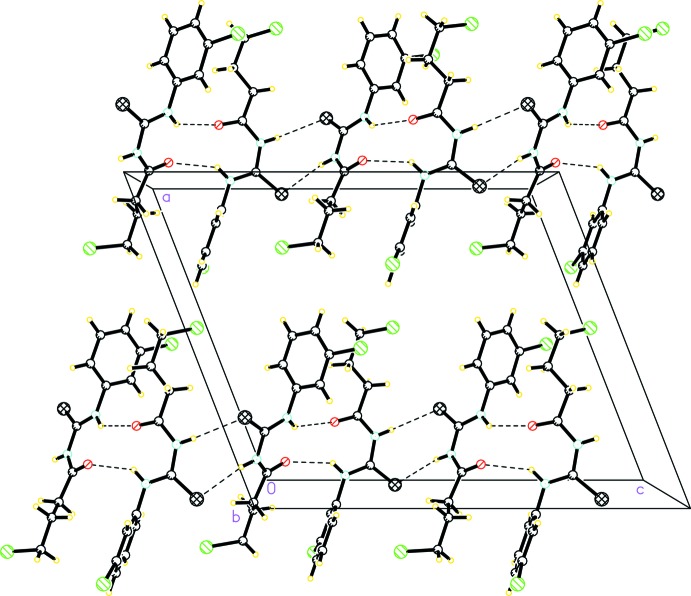
The crystal packing of the title compound viewed down the b axis. The dashes lines indicate hydrogen bonds.

## supplementary crystallographic information

### 1. Comment

Halogeno-carbonoylthiourea derivatives are useful starting materials for the synthesis of other derivatives through the reactivity of the C-halogen bonds. It is also known that the compounds are an important class of organic compounds due to their potential application as ligands and to their biological activities (Abbas *et al.*, 2013).

The title compound (Fig. 1) is an isomer of the previously reported compound 1-(4-chlorobutanoyl)-3-(2-chlorophenyl)thiourea (Yusof *et al.*, 2012) where the chlorine atom is at position-3 of the phenyl group. The asymmetric unit consists of two crystallographically independent molecules. Both molecules maintain the *trans*-*cis* configuration with respect to the position of the 4-chlorobutanoyl and 3-chlorophenyl groups, respectively, relative to the thiono S1 and S2 atoms across their C—N bonds. The thiourea moities, (N1/N2/C5/S1/C6 and N3/N4/C15/S2/C16) and benzene rings, (C6—C11 and C17–C22) in both molecules are planar with a maximum deviation of 0.023 (3) Å for atom N2. In one molecule, the benzene ring is almost perpendicular to the thiourea moiety with a dihedral angle of 87.40 (18), while in the other molecule the dihedral angle is 69.42 (15)°. Bond lengths and angles are in normal ranges (Allen *et al.*, 1987) and comparable to those in found 1-(4-chlorobutanoyl)-3-(2-chlorophenyl)thiourea. There are intramolecular hydrogen bonds between the carbonyl oxygen atom and thioamide hydrogen atom, N2—H2··· O1 and N4—H4···O2 in both molecules (Table 1). In addition, the two molecules are connected by N2—H2···O2 and N4—H4···O1 hydrogen bonds. In the crystal packing the molecules are linked by N—H···O, N—H···S and C—H···S hydrogen bonds to form one-dimensional chains along the *c* axis (Fig. 2).

### 2. Experimental

An acetone solution (30 mL) of 3-chloroaniline (0.01 mol, 1.27 g m) was added dropwise into a two-necked round-bottomed flask containing an equimolar amount of 4-chlorobutanoylisothiocyanate (0.01 mol, 1.636 g m). The mixture was refluxed for about 4 h, filtered into a beaker and left to evaporate at room temperature. The filtrate gave colourless crystals after 7 days on slow evaporation of the solvent (yield 78%).

### 3. Refinement

H atoms were positioned geometrically with C—H = 0.93–0.97 Å and constrained to ride on their parent atoms with *U*_iso_(H) = 1.2*U*_eq_(C). The H atoms on the nitrogen atoms were located in difference Fourier map and refined isotropically with the N—H distances constrained to be 0.88 (1) Å. The highest peak and deepest hole are located at 0.73 Å from atom H10 and 0.74 Å from atom Cl2, respectively. One outlier (2 0 0) was omitted from the last cycles of refinement.

### Crystal data

**Table:**

C_11_H_12_Cl_2_N_2_OS	*F*(000) = 1200
*M**_r_* = 291.19	*D*_x_ = 1.442 Mg m^−^^3^
Monoclinic, *P*2_1_/*c*	Mo *K*α radiation, λ = 0.71073 Å
Hall symbol: -P 2ybc	Cell parameters from 9934 reflections
*a* = 14.7762 (8) Å	θ = 2.9–25.5°
*b* = 10.9400 (6) Å	µ = 0.62 mm^−^^1^
*c* = 17.8153 (10) Å	*T* = 296 K
β = 111.327 (2)°	Block, colourless
*V* = 2682.7 (3) Å^3^	0.41 × 0.35 × 0.30 mm
*Z* = 8	

### Data collection

**Table:**

Bruker SMART APEX CCD area-detector diffractometer	4987 independent reflections
Radiation source: fine-focus sealed tube	3897 reflections with *I* > 2σ(*I*)
Graphite monochromator	*R*_int_ = 0.046
Detector resolution: 83.66 pixels mm^-1^	θ_max_ = 25.5°, θ_min_ = 2.9°
ω scan	*h* = −17→17
Absorption correction: multi-scan (*SADABS*; Bruker, 2009)	*k* = −13→13
*T*_min_ = 0.784, *T*_max_ = 0.835	*l* = −21→21
49874 measured reflections	

### Refinement

**Table:**

Refinement on *F*^2^	Primary atom site location: structure-invariant direct methods
Least-squares matrix: full	Secondary atom site location: difference Fourier map
*R*[*F*^2^ > 2σ(*F*^2^)] = 0.058	Hydrogen site location: inferred from neighbouring sites
*wR*(*F*^2^) = 0.162	H atoms treated by a mixture of independent and constrained refinement
*S* = 1.05	*w* = 1/[σ^2^(*F*_o_^2^) + (0.0699*P*)^2^ + 5.078*P*] where *P* = (*F*_o_^2^ + 2*F*_c_^2^)/3
4987 reflections	(Δ/σ)_max_ = 0.001
323 parameters	Δρ_max_ = 1.51 e Å^−^^3^
4 restraints	Δρ_min_ = −0.83 e Å^−^^3^

### Special details

**Table:**

Geometry. All e.s.d.'s (except the e.s.d. in the dihedral angle between two l.s. planes) are estimated using the full covariance matrix. The cell e.s.d.'s are taken into account individually in the estimation of e.s.d.'s in distances, angles and torsion angles; correlations between e.s.d.'s in cell parameters are only used when they are defined by crystal symmetry. An approximate (isotropic) treatment of cell e.s.d.'s is used for estimating e.s.d.'s involving l.s. planes.
Refinement. Refinement of *F*^2^ against ALL reflections. The weighted *R*-factor *wR* and goodness of fit *S* are based on *F*^2^, conventional *R*-factors *R* are based on *F*, with *F* set to zero for negative *F*^2^. The threshold expression of *F*^2^ > σ(*F*^2^) is used only for calculating *R*-factors(gt) *etc*. and is not relevant to the choice of reflections for refinement. *R*-factors based on *F*^2^ are statistically about twice as large as those based on *F*, and *R*- factors based on ALL data will be even larger.

### Fractional atomic coordinates and isotropic or equivalent isotropic displacement parameters (Å^2^)

**Table:**

	*x*	*y*	*z*	*U*_iso_*/*U*_eq_	
Cl1	1.20900 (11)	0.68433 (14)	0.71321 (7)	0.0842 (4)	
Cl2	0.53415 (11)	1.04818 (15)	0.11355 (8)	0.0871 (4)	
Cl3	0.47334 (9)	0.68932 (12)	0.00986 (9)	0.0842 (4)	
Cl4	1.26802 (10)	0.33013 (12)	0.45687 (9)	0.0839 (4)	
S1	0.75626 (6)	1.01805 (8)	0.46585 (5)	0.0406 (2)	
S2	1.01747 (6)	0.48468 (9)	0.16368 (5)	0.0397 (2)	
O1	0.92224 (18)	0.6927 (2)	0.42275 (15)	0.0509 (7)	
O2	0.79236 (17)	0.6402 (3)	0.25577 (15)	0.0571 (8)	
N1	0.90164 (18)	0.8658 (3)	0.48549 (16)	0.0327 (6)	
N2	0.7673 (2)	0.8405 (3)	0.36889 (17)	0.0400 (7)	
N3	0.84653 (19)	0.5491 (3)	0.16566 (16)	0.0355 (6)	
N4	0.97653 (19)	0.5835 (3)	0.28377 (17)	0.0405 (7)	
C1	1.1947 (3)	0.6227 (4)	0.6168 (2)	0.0580 (11)	
H1A	1.2353	0.6683	0.5944	0.070*	
H1B	1.2167	0.5385	0.6233	0.070*	
C2	1.0906 (3)	0.6275 (3)	0.5587 (2)	0.0433 (8)	
H2A	1.0863	0.5891	0.5084	0.052*	
H2B	1.0502	0.5812	0.5809	0.052*	
C3	1.0526 (2)	0.7563 (3)	0.5420 (2)	0.0429 (8)	
H3A	1.0506	0.7913	0.5914	0.051*	
H3B	1.0974	0.8046	0.5258	0.051*	
C4	0.9534 (2)	0.7651 (3)	0.4779 (2)	0.0362 (7)	
C5	0.8088 (2)	0.9018 (3)	0.43661 (18)	0.0325 (7)	
C6	0.6695 (3)	0.8650 (4)	0.3146 (2)	0.0428 (9)	
C7	0.6542 (3)	0.9400 (4)	0.2506 (2)	0.0484 (9)	
H7	0.7055	0.9786	0.2417	0.058*	
C8	0.5567 (3)	0.9572 (4)	0.1977 (2)	0.0543 (10)	
C9	0.4823 (3)	0.9004 (5)	0.2106 (3)	0.0635 (12)	
H9	0.4190	0.9126	0.1749	0.076*	
C10	0.4990 (3)	0.8265 (5)	0.2745 (3)	0.0674 (13)	
H10	0.4475	0.7882	0.2832	0.081*	
C11	0.5940 (3)	0.8079 (4)	0.3275 (2)	0.0567 (11)	
H11	0.6062	0.7566	0.3717	0.068*	
C12	0.4953 (3)	0.6064 (5)	0.1009 (3)	0.0606 (11)	
H12A	0.4501	0.6334	0.1256	0.073*	
H12B	0.4838	0.5201	0.0885	0.073*	
C13	0.5976 (2)	0.6237 (4)	0.1596 (2)	0.0523 (10)	
H13A	0.6107	0.7106	0.1674	0.063*	
H13B	0.6027	0.5891	0.2111	0.063*	
C14	0.6733 (2)	0.5662 (3)	0.1331 (2)	0.0383 (8)	
H14A	0.6624	0.4787	0.1278	0.046*	
H14B	0.6668	0.5981	0.0806	0.046*	
C15	0.7744 (2)	0.5901 (3)	0.1911 (2)	0.0376 (8)	
C16	0.9453 (2)	0.5431 (3)	0.20854 (18)	0.0310 (7)	
C17	1.0758 (2)	0.5800 (3)	0.33715 (18)	0.0330 (7)	
C18	1.1187 (2)	0.4690 (3)	0.3653 (2)	0.0385 (8)	
H18	1.0850	0.3961	0.3481	0.046*	
C19	1.2132 (3)	0.4690 (3)	0.4198 (2)	0.0414 (8)	
C20	1.2647 (2)	0.5753 (4)	0.4454 (2)	0.0473 (9)	
H20	1.3286	0.5732	0.4816	0.057*	
C21	1.2201 (3)	0.6843 (4)	0.4166 (3)	0.0521 (10)	
H21	1.2539	0.7571	0.4336	0.062*	
C22	1.1247 (3)	0.6873 (3)	0.3621 (2)	0.0426 (8)	
H22	1.0945	0.7615	0.3429	0.051*	
H1	0.923 (2)	0.912 (3)	0.5285 (13)	0.033 (9)*	
H3	0.826 (2)	0.521 (3)	0.1164 (9)	0.030 (9)*	
H4	0.933 (2)	0.609 (3)	0.303 (2)	0.043 (10)*	
H2	0.801 (3)	0.779 (3)	0.360 (2)	0.053 (12)*	

### Atomic displacement parameters (Å^2^)

**Table:**

	*U*^11^	*U*^22^	*U*^33^	*U*^12^	*U*^13^	*U*^23^
Cl1	0.0966 (10)	0.0931 (9)	0.0404 (6)	0.0185 (8)	−0.0019 (6)	−0.0008 (6)
Cl2	0.0826 (9)	0.1067 (11)	0.0642 (7)	0.0309 (8)	0.0174 (7)	0.0311 (7)
Cl3	0.0508 (6)	0.0749 (8)	0.0930 (9)	0.0089 (6)	−0.0142 (6)	0.0095 (7)
Cl4	0.0744 (8)	0.0630 (7)	0.0882 (9)	0.0298 (6)	−0.0016 (7)	0.0157 (6)
S1	0.0343 (4)	0.0466 (5)	0.0349 (4)	0.0111 (4)	0.0057 (3)	−0.0043 (4)
S2	0.0316 (4)	0.0535 (5)	0.0337 (4)	0.0046 (4)	0.0116 (3)	−0.0006 (4)
O1	0.0388 (14)	0.0542 (16)	0.0470 (15)	0.0099 (12)	0.0005 (12)	−0.0182 (13)
O2	0.0313 (13)	0.087 (2)	0.0415 (14)	0.0122 (13)	−0.0002 (11)	−0.0240 (14)
N1	0.0259 (13)	0.0376 (15)	0.0301 (14)	0.0035 (11)	0.0048 (11)	−0.0062 (12)
N2	0.0285 (14)	0.0493 (18)	0.0343 (15)	0.0103 (13)	0.0018 (12)	−0.0088 (13)
N3	0.0247 (13)	0.0504 (17)	0.0265 (14)	0.0015 (12)	0.0036 (11)	−0.0055 (12)
N4	0.0257 (14)	0.0582 (19)	0.0324 (15)	0.0087 (13)	0.0042 (12)	−0.0090 (13)
C1	0.048 (2)	0.070 (3)	0.046 (2)	0.022 (2)	0.0051 (18)	0.001 (2)
C2	0.0375 (19)	0.047 (2)	0.0403 (19)	0.0074 (16)	0.0084 (15)	−0.0009 (16)
C3	0.0273 (16)	0.045 (2)	0.047 (2)	0.0056 (15)	0.0021 (15)	−0.0064 (16)
C4	0.0287 (16)	0.0417 (19)	0.0355 (17)	0.0030 (14)	0.0086 (14)	−0.0005 (15)
C5	0.0275 (15)	0.0380 (17)	0.0305 (16)	0.0012 (13)	0.0087 (13)	0.0049 (14)
C6	0.0376 (19)	0.051 (2)	0.0306 (17)	0.0129 (16)	0.0015 (14)	−0.0097 (16)
C7	0.046 (2)	0.055 (2)	0.039 (2)	0.0085 (18)	0.0091 (16)	−0.0054 (17)
C8	0.056 (2)	0.059 (2)	0.040 (2)	0.016 (2)	0.0090 (18)	0.0009 (18)
C9	0.037 (2)	0.084 (3)	0.059 (3)	0.010 (2)	0.0051 (19)	−0.007 (2)
C10	0.034 (2)	0.097 (4)	0.062 (3)	−0.001 (2)	0.007 (2)	−0.005 (3)
C11	0.038 (2)	0.077 (3)	0.047 (2)	0.001 (2)	0.0051 (17)	−0.003 (2)
C12	0.0295 (19)	0.080 (3)	0.068 (3)	−0.0057 (19)	0.0134 (19)	−0.016 (2)
C13	0.0291 (18)	0.075 (3)	0.051 (2)	−0.0026 (18)	0.0115 (16)	−0.015 (2)
C14	0.0277 (16)	0.046 (2)	0.0366 (18)	0.0010 (14)	0.0056 (14)	−0.0046 (15)
C15	0.0281 (16)	0.0451 (19)	0.0349 (18)	0.0054 (14)	0.0058 (14)	−0.0021 (15)
C16	0.0264 (15)	0.0327 (16)	0.0300 (16)	0.0016 (12)	0.0055 (13)	0.0039 (13)
C17	0.0257 (15)	0.0437 (19)	0.0269 (15)	0.0046 (14)	0.0065 (13)	−0.0032 (14)
C18	0.0343 (17)	0.0391 (18)	0.0373 (18)	−0.0011 (14)	0.0073 (14)	−0.0051 (15)
C19	0.0353 (18)	0.048 (2)	0.0374 (18)	0.0128 (16)	0.0088 (15)	0.0022 (16)
C20	0.0242 (16)	0.069 (3)	0.042 (2)	0.0025 (17)	0.0035 (14)	−0.0063 (18)
C21	0.037 (2)	0.050 (2)	0.061 (2)	−0.0093 (17)	0.0092 (18)	−0.0112 (19)
C22	0.0369 (18)	0.0385 (19)	0.047 (2)	0.0044 (15)	0.0084 (16)	0.0034 (16)

### Geometric parameters (Å, º)

**Table:**

Cl1—C1	1.784 (4)	C6—C7	1.357 (5)
Cl2—C8	1.728 (4)	C6—C11	1.369 (6)
Cl3—C12	1.782 (5)	C7—C8	1.418 (5)
Cl4—C19	1.736 (4)	C7—H7	0.9300
S1—C5	1.669 (3)	C8—C9	1.354 (6)
S2—C16	1.674 (3)	C9—C10	1.343 (7)
O1—C4	1.215 (4)	C9—H9	0.9300
O2—C15	1.215 (4)	C10—C11	1.393 (6)
N1—C4	1.376 (4)	C10—H10	0.9300
N1—C5	1.388 (4)	C11—H11	0.9300
N1—H1	0.876 (10)	C12—C13	1.504 (5)
N2—C5	1.321 (4)	C12—H12A	0.9700
N2—C6	1.441 (4)	C12—H12B	0.9700
N2—H2	0.876 (10)	C13—C14	1.502 (5)
N3—C15	1.376 (4)	C13—H13A	0.9700
N3—C16	1.381 (4)	C13—H13B	0.9700
N3—H3	0.872 (10)	C14—C15	1.497 (4)
N4—C16	1.325 (4)	C14—H14A	0.9700
N4—C17	1.429 (4)	C14—H14B	0.9700
N4—H4	0.874 (10)	C17—C22	1.365 (5)
C1—C2	1.511 (5)	C17—C18	1.378 (5)
C1—H1A	0.9700	C18—C19	1.381 (5)
C1—H1B	0.9700	C18—H18	0.9300
C2—C3	1.506 (5)	C19—C20	1.373 (5)
C2—H2A	0.9700	C20—C21	1.369 (6)
C2—H2B	0.9700	C20—H20	0.9300
C3—C4	1.499 (4)	C21—C22	1.391 (5)
C3—H3A	0.9700	C21—H21	0.9300
C3—H3B	0.9700	C22—H22	0.9300
			
C4—N1—C5	128.6 (3)	C9—C10—C11	119.5 (4)
C4—N1—H1	121 (2)	C9—C10—H10	120.3
C5—N1—H1	110 (2)	C11—C10—H10	120.3
C5—N2—C6	122.5 (3)	C6—C11—C10	120.1 (4)
C5—N2—H2	116 (3)	C6—C11—H11	120.0
C6—N2—H2	121 (3)	C10—C11—H11	120.0
C15—N3—C16	128.5 (3)	C13—C12—Cl3	111.9 (3)
C15—N3—H3	115 (2)	C13—C12—H12A	109.2
C16—N3—H3	117 (2)	Cl3—C12—H12A	109.2
C16—N4—C17	124.0 (3)	C13—C12—H12B	109.2
C16—N4—H4	118 (2)	Cl3—C12—H12B	109.2
C17—N4—H4	118 (2)	H12A—C12—H12B	107.9
C2—C1—Cl1	112.4 (3)	C14—C13—C12	113.8 (3)
C2—C1—H1A	109.1	C14—C13—H13A	108.8
Cl1—C1—H1A	109.1	C12—C13—H13A	108.8
C2—C1—H1B	109.1	C14—C13—H13B	108.8
Cl1—C1—H1B	109.1	C12—C13—H13B	108.8
H1A—C1—H1B	107.9	H13A—C13—H13B	107.7
C3—C2—C1	112.4 (3)	C15—C14—C13	112.4 (3)
C3—C2—H2A	109.1	C15—C14—H14A	109.1
C1—C2—H2A	109.1	C13—C14—H14A	109.1
C3—C2—H2B	109.1	C15—C14—H14B	109.1
C1—C2—H2B	109.1	C13—C14—H14B	109.1
H2A—C2—H2B	107.9	H14A—C14—H14B	107.9
C4—C3—C2	113.7 (3)	O2—C15—N3	122.0 (3)
C4—C3—H3A	108.8	O2—C15—C14	123.5 (3)
C2—C3—H3A	108.8	N3—C15—C14	114.4 (3)
C4—C3—H3B	108.8	N4—C16—N3	116.9 (3)
C2—C3—H3B	108.8	N4—C16—S2	124.2 (2)
H3A—C3—H3B	107.7	N3—C16—S2	118.8 (2)
O1—C4—N1	122.8 (3)	C22—C17—C18	121.4 (3)
O1—C4—C3	123.5 (3)	C22—C17—N4	119.2 (3)
N1—C4—C3	113.7 (3)	C18—C17—N4	119.4 (3)
N2—C5—N1	117.0 (3)	C17—C18—C19	118.0 (3)
N2—C5—S1	123.9 (2)	C17—C18—H18	121.0
N1—C5—S1	119.1 (2)	C19—C18—H18	121.0
C7—C6—C11	121.4 (3)	C20—C19—C18	122.1 (3)
C7—C6—N2	119.8 (4)	C20—C19—Cl4	119.3 (3)
C11—C6—N2	118.8 (3)	C18—C19—Cl4	118.7 (3)
C6—C7—C8	117.2 (4)	C21—C20—C19	118.7 (3)
C6—C7—H7	121.4	C21—C20—H20	120.7
C8—C7—H7	121.4	C19—C20—H20	120.7
C9—C8—C7	121.2 (4)	C20—C21—C22	120.6 (4)
C9—C8—Cl2	120.0 (3)	C20—C21—H21	119.7
C7—C8—Cl2	118.8 (4)	C22—C21—H21	119.7
C10—C9—C8	120.7 (4)	C17—C22—C21	119.3 (3)
C10—C9—H9	119.7	C17—C22—H22	120.3
C8—C9—H9	119.7	C21—C22—H22	120.3
			
Cl1—C1—C2—C3	61.8 (4)	Cl3—C12—C13—C14	−68.4 (5)
C1—C2—C3—C4	173.7 (3)	C12—C13—C14—C15	177.4 (4)
C5—N1—C4—O1	1.6 (6)	C16—N3—C15—O2	8.2 (6)
C5—N1—C4—C3	179.5 (3)	C16—N3—C15—C14	−171.1 (3)
C2—C3—C4—O1	−28.8 (5)	C13—C14—C15—O2	6.6 (5)
C2—C3—C4—N1	153.3 (3)	C13—C14—C15—N3	−174.1 (3)
C6—N2—C5—N1	177.1 (3)	C17—N4—C16—N3	177.8 (3)
C6—N2—C5—S1	−2.8 (5)	C17—N4—C16—S2	−1.4 (5)
C4—N1—C5—N2	−8.1 (5)	C15—N3—C16—N4	−1.2 (5)
C4—N1—C5—S1	171.8 (3)	C15—N3—C16—S2	178.0 (3)
C5—N2—C6—C7	95.8 (4)	C16—N4—C17—C22	113.8 (4)
C5—N2—C6—C11	−86.6 (5)	C16—N4—C17—C18	−69.3 (5)
C11—C6—C7—C8	0.2 (6)	C22—C17—C18—C19	0.0 (5)
N2—C6—C7—C8	177.8 (3)	N4—C17—C18—C19	−176.9 (3)
C6—C7—C8—C9	−0.3 (6)	C17—C18—C19—C20	−0.8 (5)
C6—C7—C8—Cl2	−178.0 (3)	C17—C18—C19—Cl4	179.3 (3)
C7—C8—C9—C10	0.4 (7)	C18—C19—C20—C21	1.0 (6)
Cl2—C8—C9—C10	178.0 (4)	Cl4—C19—C20—C21	−179.1 (3)
C8—C9—C10—C11	−0.4 (7)	C19—C20—C21—C22	−0.4 (6)
C7—C6—C11—C10	−0.2 (6)	C18—C17—C22—C21	0.5 (5)
N2—C6—C11—C10	−177.8 (4)	N4—C17—C22—C21	177.4 (3)
C9—C10—C11—C6	0.3 (7)	C20—C21—C22—C17	−0.3 (6)

### Hydrogen-bond geometry (Å, º)

**Table:**

*D*—H···*A*	*D*—H	H···*A*	*D*···*A*	*D*—H···*A*
N2—H2···O1	0.89 (4)	1.97 (4)	2.679 (4)	136 (3)
N2—H2···O2	0.89 (4)	2.37 (3)	3.089 (4)	139 (3)
N4—H4···O1	0.88 (3)	2.38 (3)	3.106 (4)	140 (3)
N4—H4···O2	0.88 (3)	1.97 (3)	2.656 (4)	134 (3)
C3—H3*A*···Cl1	0.97	2.80	3.184 (4)	104
N1—H1···S2^i^	0.88 (3)	2.57 (2)	3.425 (3)	167 (3)
N3—H3···S1^ii^	0.87 (2)	2.54 (2)	3.397 (3)	169 (3)
C3—H3*B*···S1^iii^	0.97	2.87	3.792 (3)	160

Symmetry codes: (i) *x*, −*y*+3/2, *z*+1/2; (ii) *x*, −*y*+3/2, *z*−1/2; (iii) −*x*+2, −*y*+2, −*z*+1.
